# Harnessing agro-waste for the high-efficiency removal of methylene blue using ball-milled magnetic Fe_3_O_4_@pistachio shell composites: From waste to resource

**DOI:** 10.1371/journal.pone.0337235

**Published:** 2025-11-25

**Authors:** Tamer S. Saleh, Mohamed N. Gomaa, Abdullah Akhdhar, Abdullah S. Al‐Bogami, Waleed A. El-Said

**Affiliations:** 1 Department of Chemistry, College of Science, University of Jeddah, Jeddah, Saudi Arabia; 2 Department of Biology Science, College of Science, University of Jeddah, Jeddah, Saudi Arabia; 3 Department of Chemistry, Faculty of Science, Assiut University, Assiut, Egypt; King Saud University, SAUDI ARABIA

## Abstract

The development of a cost-effective and ecologically beneficial method for removing toxic dye molecules from wastewater is urgently needed for environmental and health reasons. Here, four Fe_3_O_4_/PS nanocomposites with different Fe_3_O_4_ percentages were fabricated through the mechanochemical technique. This study provided a new monolithic adsorbent from abundant materials via a facile synthetic procedure, which can greatly reduce the problems of the tedious separation of adsorbents from treated waste. The adsorbent is composed of pistachio shell/Fe_3_O_4_ composite, which has been used as a highly effective and sustainable adsorbent to eliminate methylene blue (MB) dye under ambient conditions. Diverse characterization analyses, including SEM, EDX, and FTIR techniques, were utilized to investigate the morphology and structure of the synthesized composite. The formed Fe_3_O_4_ particles have an average diameter of 274 nm and an average area of 0.11 μm^2^. The batch adsorption methodology was employed, wherein several parameters, such as adsorbent dose, pH, initial dye concentration, adsorption temperature, and contact time, were adjusted to examine their effectiveness and adsorption capacity in removing the MB dye. Furthermore, the adsorption behavior was evaluated by studying the linear and non-linear adsorption isotherms. The maximum MB removal efficiency of 95% was achieved with the optimized parameters of MB dye concentration (2.5 mg/L), adsorbent dose (15 mg), contact time (15 min), and adsorption temperature (25 °C). The selectivity of the developed adsorbent was examined towards 25 mg/L of MB cationic dye and anionic dye (methylene orange), which demonstrated higher removal efficiency for the cationic dye (46.93%) compared to the anionic dye (12.46%). Moreover, the pistachio shell/Fe_3_O_4_ adsorbent demonstrated excellent capability to remove MB dye from industrial wastewater samples. Overall, this approach presents a new, sustainable, and effective strategy for mitigating the harmful effects of MB, with potential applications in treated industrial wastewater samples.

## 1. Introduction

Chemical dyes are among the industrial water pollutants posing a serious environmental hazard. The presence of synthetic dyes, such as methylene blue (MB), in water bodies presents a significant challenge to both the environment and public health due to their toxic nature, persistence, and resistance to standard wastewater treatment techniques [1–4]. MB, a cationic dye commonly used in the textile, paper, and pharmaceutical sectors, frequently infiltrates aquatic systems through industrial effluents. This intrusion has harmful consequences for aquatic organisms and human health by diminishing light penetration and oxygen levels in water [5,6]. Consequently, it is essential to develop effective, economical, and sustainable methods for removing MB from polluted water.

Adsorption has gained recognition for its effectiveness in removing heavy metals and dyes, characterized by straightforward application, high efficiency, cost-effectiveness, and minimal environmental impact [7–13]. This technique allows dye molecules to migrate from the liquid phase to the surface of the solid adsorbents, therefore enabling simple separation and regeneration of the materials [14–16]. Agricultural waste materials have gained great attention among several adsorbents because of their abundance, renewability, sustainability, and natural adsorption characteristics arising from their lignocellulosic composition and surface functional groups [17,18]. Often needing physicochemical changes to improve their adsorption capability, surface area, and selectivity for dye compounds, unprocessed agricultural waste [19]. An affordable biosorbent for color removal, a by-product of agro-industrial operations, pistachio shell (PS) has shown great potential [20]. A significant portion of agro-industrial waste generated worldwide, PS is commonly burned or thrown away, thus causing resource waste and environmental damage [21]. Including lignocellulosic biomass rich in cellulose, hemicellulose, and lignin in adsorbent composites increases value from an otherwise low-value byproduct, thereby supporting circular economy goals and waste minimization [22,23]. Studies have demonstrated that PS composites improve biodegradability, mechanical stability, and exhibit enhanced adsorption of organic dyes, attributable to interactions between surface functional groups and dye molecules [23]. PS powder is renewable and biodegradable, thereby providing a green substitute for synthetic fillers and adsorbents. Using PS as the main component in the composite to eliminate MB greatly conforms to the sustainability values. Studies show that composites using PS powder demonstrate improved biodegradability; they naturally break down through microbial activity without producing damaging residues, thereby lowering the environmental impact following use [24]. The noted increases in the mechanical and thermal stabilities of PS-based composites support their durability and useful applications in water treatment technologies being. With the adsorption processes mostly physical and endothermic, research has demonstrated that chemically treated pistachio shell powder exhibits better adsorption of methylene blue due to interactions between surface acidic oxygen groups and dye nitrogen atoms. To increase practicality, magnetic nanoparticles such as Fe₃O₄ have been incorporated into PS to impart magnetic properties, allowing simple separation via external magnetic fields and reuse of adsorbents, while enhancing surface area and active site availability [25]. Apart from improving the effectiveness of pollution removal, the magnetic Fe₃O₄ functionalization helps simple adsorbent recovery and reuse, so reducing secondary waste and running costs [26,27]. Compared to typical filtration techniques, this magnetic separation capacity lowers energy consumption, therefore reducing the total environmental impact of the water treatment process. Recent work has concentrated on building magnetic composites with iron oxide nanoparticles and PS biochar integration to achieve high methylene blue removal efficiency. These composites indicated a considerable regeneration potential by displaying promising adsorption kinetics and isotherms, therefore attaining maximum removal efficiencies larger than 99% under optimal conditions. While also enabling the quick and efficient recovery of the adsorbent, therefore addressing common problems in dye wastewater treatment, the addition of Fe₃O₄ greatly increases the adsorption capacities by increasing the surface area and active sites [28,29].

Despite these advancements, gaps remain in optimizing fabrication methods such as mechanochemical ball milling for Fe₃O₄/PS composites, systematically evaluating adsorption parameters, elucidating adsorption mechanisms under varying conditions, assessing selectivity towards cationic versus anionic dyes, and testing durability through reusability and industrial wastewater treatment.

Bearing in mind all the above-mentioned and in line with our continuous investigation into offering creatively sustainable economic methods for water treatment from dyes [30–35]. This study addresses the present gaps by developing and characterizing ball-milled Fe₃O₄/PS nanocomposites with variable Fe₃O₄ loadings, investigating adsorption kinetics and isotherms, optimizing operational parameters, and evaluating adsorbent selectivity and regeneration performance. Such an approach offers a sustainable, economic, and effective strategy for wastewater remediation by harnessing agro-waste materials.

We developed several adsorbents, including PS, Fe_3_O_4_, and four different PS/Fe_3_O_4_ composites for MB removal. The Fe_3_O_4_/PS composites were fabricated using the mechanochemical method. The fabricated adsorbents were characterized by various techniques, including SEM, EDX, and FTIR. Notably, this study represents the combination of the Fe_3_O_4_ nanoparticles, including their high magnetic properties and their negatively charged surface, which leads to easy magnetic recovery, with the low cost and sustainability of the PS. The adsorbents show high removal efficiency and selectivity toward MB in the presence of anionic dye and industrial wastewater samples. Furthermore, the Fe_3_O_4_/PS adsorbents demonstrate high stability and regeneration ability.

## 2. Materials and methods

### 2.1. Materials

Cellulosic waste was obtained from the Saudi market and taken for grinding into fine powder via a ball mill utilizing three balls with a diameter of 16 mm to obtain fine powder. Methylene blue (**1**, high purity, biological stain), ferric chloride (FeCl_3_), sodium hydroxide (NaOH), and potassium iodide (KI) were supplied by Sigma-Aldrich.

### 2.2. Synthesis of iron oxide and iron oxide/PS composite

A 0.12 mol of FeCl_3_ was dissolved in 150 mL of distilled water and mixed with a 50 mL aqueous solution of 0.04 mol of KI at room temperature. The mixture was stirred for one hour. The reaction mixture was filtered. The filtrate was hydrolyzed by dropwise adding 0.1 mol/L NaOH with continuous stirring until pH 9–11. The reaction was then left to settle for 24 h. The reaction was filtered, and the black precipitate was washed with distilled water and dried in an oven at 80 °C for 6 hrs. PS powder was obtained by using a ball mill. Different Fe_3_O_4_/PS composites were obtained by mixing different percentages of Fe_3_O_4_ with PS powder. Then, Fe_3_O_4_ and PS powder were allowed to form the composite by using a ball mill for 10 min.

### 2.3. Dye adsorption studies

A stock solution of MB dye in water (100 mg L^-1^) was prepared and diluted to various concentrations as required. The removal experiments were carried out by mixing the adsorbent with the aqueous dye solution (25 mL) and stirring at 298 K. After an appropriate time, the dye solution was filtered and analyzed by UV–vis spectrophotometry over a wavelength range from 200 nm to 700 nm.

## 3. Results and discussion

### 3.1. Synthesis, optimization, and characterization of Fe_3_O_4_/pistachio nanocomposites

**Fig 1:**
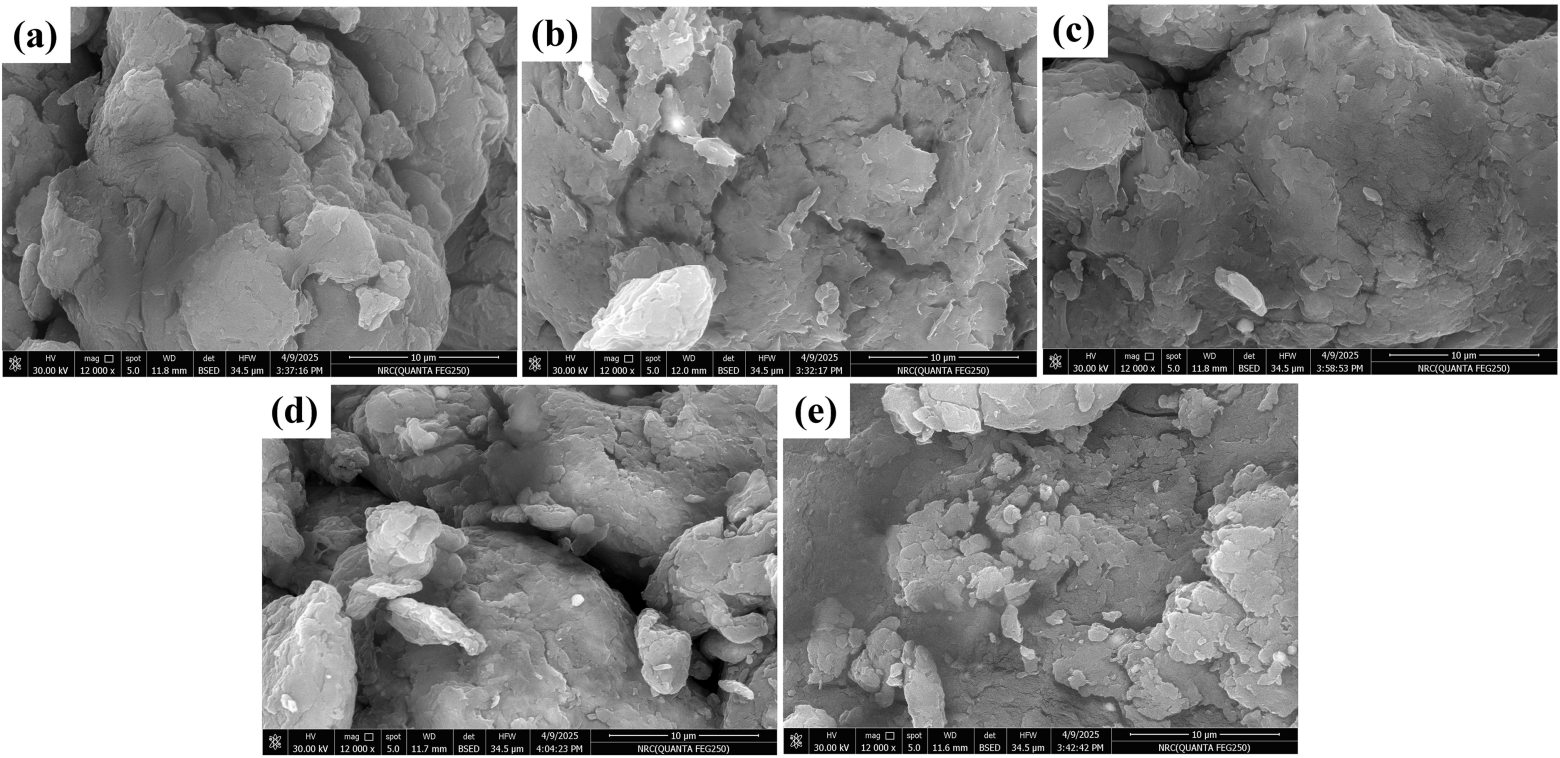
SEM image of (a) PS powder, (b) 5% Fe_3_O_4_/PS composite, (c) 10% Fe_3_O_4_/PS composite, (d) 20% Fe_3_O_4_/PS composite, and (e) 30% Fe_3_O_4_/PS composite. The SEM images were recorded at magnification power of 12 KX.

Different Fe_3_O_4_/pistachio nanocomposites were fabricated by varying the Fe_3_O_4_ content from 5 to 30 wt.%. The composites were then obtained by using a ball mill for 15 min. The morphology and chemical compositions of the developed composites were investigated through the SEM, FTIR, XRD, BET, Zeta potential, and EDX techniques. **Fig 1a** shows the SEM image of the pure pistachio powder; the morphology demonstrates the presence of large flakes. The morphologies of the different Fe_3_O_4_/pistachio nanocomposites were examined using SEM observation. **Fig 1b**–e show the SEM images of the Fe_3_O_4_/pistachio nanocomposites, which demonstrate distinct differences between the bare pistachio shell powder and the Fe₃O₄/pistachio shell nanocomposites. The pistachio shell powder displays large, irregular flakes with a rough and porous surface structure, characteristic of lignocellulosic biomass. Upon incorporation of Fe₃O₄ nanoparticles, the surface of the composites shows the appearance of fine, uniformly dispersed nanoparticles anchored on the biomaterial matrix. Image analysis confirms that the Fe₃O₄ particles have an average diameter of 274 nm, and their distribution increases with greater iron oxide content, as evidenced by EDX spectra. At higher Fe₃O₄ loading, the nanoparticles tend to cover more surface area, enhancing the composite’s overall roughness and increasing the available active sites for adsorption. However, excessive Fe₃O₄ may block essential pores, slightly reducing adsorption performance, as seen for the 30% composite.

Furthermore, the Fe_3_O_4_ particle area and diameter are studied by analyzing the SEM image using ImageJ software (S1 and S2 Figs in S1 File). S1 Fig in S1 File demonstrates the diameter distribution of 45 particles, which were randomly selected. The results show that the average diameter of the Fe_3_O_4_ particles is 274 nm with a standard deviation (SD) of 89.9 nm. Also, the area distribution of Fe_3_O_4_ particles was analyzed as shown in S2 Fig in S1 File. The result reveals that the formed Fe_3_O_4_ particles have an average area of 0.11 μm^2^ with a SD of 0.0065 μm^2^. SEM images support the synergistic relationship between the porous structure of pistachio shell and the high surface area of Fe₃O₄ nanoparticles, which together maximize adsorption capacity and facilitate magnetic separation.

**Fig 2:**
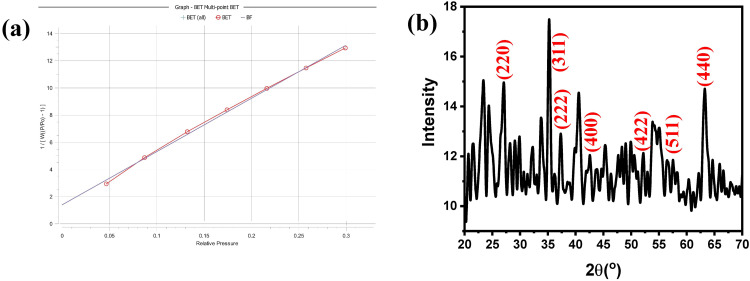
Show the BET N2 adsorption/desorption isotherm for calculating the surface area of Fe_3_O_4_ (a), and the XRD pattern of Fe_3_O_4_ (b).

The FTIR spectra of the different Fe_3_O_4_/PS nanocomposites within a wavenumber range from 400 to 4000 cm^-1^ are represented in S3–S7 Figs in S1 File. S3 Fig in S1 File displays the FTIR spectrum of the pure PS powder. The FTIR spectrum showed a set of absorption bands in the range from 600 to 900 cm^-1^ corresponding to C–H vibrations. The broadband centered at around 3355 cm^-1^ and the band at 1030 cm^-1^ are associated with hydroxyl group (–OH) vibrations (cellulose) [36]. Also, the absorption band at 1425 cm^-1^ is related to the scissoring motion of CH_2_ (cellulose). The bands at 1240 and 1506 cm^-1^ are attributed to C-O-C stretching (lignin and cellulose), and C = C bonds in aromatic rings in lignin. Furthermore, the band at 2880 cm^-1^ is attributed to the aliphatic C–H vibrations [37]. The surface area of the fabricated iron oxide nanoparticles was calculated using the BET method, as shown in **Fig 2a**, indicating the formation of iron oxide with a surface area of 85.717 m²/g, which is a good surface area for metal oxides. Furthermore, the pore volume is 0.326 cc/g, and the pore radius Dv(r) is 1.94 nm.

S4–S7 Figs in S1 File demonstrate the FTIR spectra of different Fe3O4/PS nanocomposites (5%, 10%, 20%, and 30%). The absorption bands set at 419 and those from 600 to 659 cm^-1^ are related to Fe_3_O_4_ NPs [38]. The Fe–O band was observed at 1377 cm^-1^ [39]. The broad absorption band at around 3403 cm^-1^ is attributed to O–H from adsorbed water, and the absorption band at 2881 cm^-1^ corresponds to C–H group [40–42].

The chemical composition and crystal structure of the as-prepared iron oxide NPs were studied using XRD (**Fig 2b**). The XRD pattern demonstrates a set of peaks at 2θ(°) of 27.06, 35.26, 37.33, 42.51, 56.46, and 63.27 corresponding to the lattice planes (220), (311), (400), (422), (511), and (440), respectively. These results confirm the fabrication of Fe_3_O_4_ [43]. Furthermore, the noise and low-intensity peaks indicate the amorphous nature of the synthesized material.

**Fig 3:**
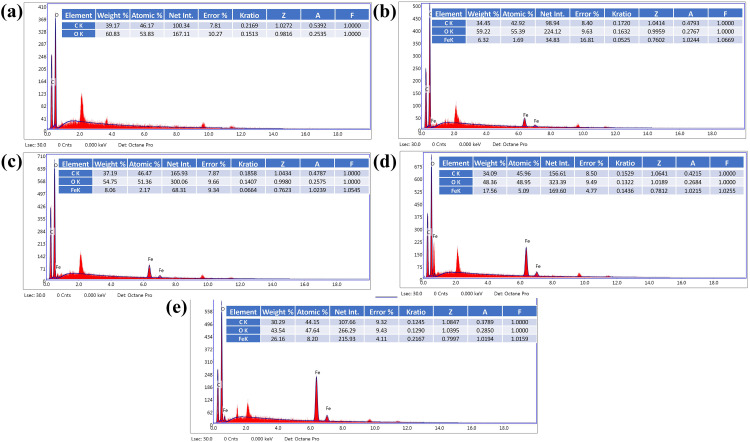
EDX of (a) PS powder, (b) 5% Fe_3_O_4_/PS composite, (c) 10% Fe_3_O_4_/PS composite, (d) 20% Fe_3_O_4_/PS composite, and (e) 30% Fe_3_O_4_/PS composite.

The EDX technique has been used to confirm the composition of the pure PS and the Fe_3_O_4_/PS nanocomposites (5%, 10%, 20%, and 30%). The EDX spectrum of the pure PS powder is shown in (**Fig 3a**). This result demonstrated that the PS powder sample contains C (39.17%) and O (60.83%) elements. Furthermore, the EDX spectra of the Fe_3_O_4_/PS nanocomposites are represented in Fig 3b–e, which show the appearance of new peaks related to the Fe element. Moreover, the results confirmed that the percentage of the Fe element increases with an increase in the iron oxide percentage from 5% to 30%.

### 3.2. Optimization of MB capture

#### 3.2.1. Adsorbent type.

Several adsorbents were developed and used for MB capture, including pure PS powder, pure Fe_3_O_4_ NPs, and four Fe_3_O_4_/PS nanocomposites (5%, 10%, 20%, and 30%). 15 mg of each adsorbent was mixed with 50 mL of a 25 mg/L MB solution. The mixtures were stirred for 15 minutes at 25 °C and then filtered.

**Fig 4:**
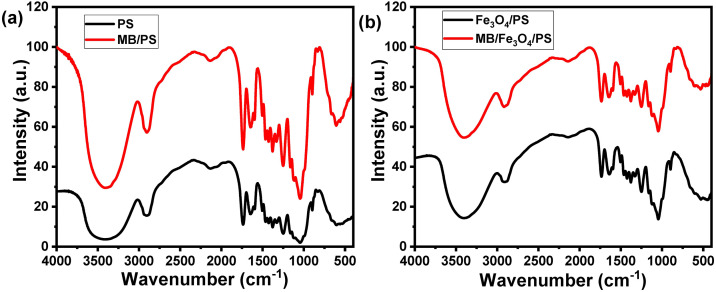
Investigation of the MB adsorption by PS and PS/Fe_3_O_4_ composites. (a) FTIR spectra of PS powder before and after MB adsorption and (b) FTIR spectra of MB before and after treatment with Fe_3_O_4_/PS composites.

Moreover, The FTIR spectra of the PS powder and Fe_3_O_4_/PS composites before and after MB adsorption were recorded (**Fig 4**). The results indicate that no obvious change occurred after the adsorption of MB on the composite’s functional groups due to the overlapping between the functional groups of MB and PS. Howere, a change in the peak intensities could be observed.

**Fig 5:**
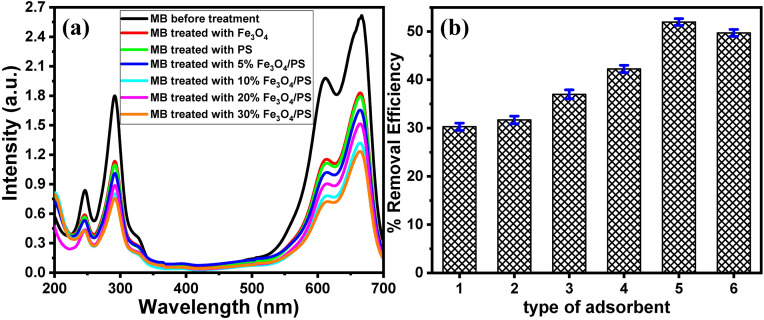
(a) UV-vis spectra of 25 mg/L of MB solution before and after treatment with Fe_3_O_4_, PS powder, and different Fe_3_O_4_/PS composites, and (b) the effect of the type of adsorbent on the removal efficiency.


**Fig 5a** shows the UV-vis spectra of 25 mg/L MB solutions before and after treatment with 15 mg of each adsorbent. The UV spectrum of the MB solution shows four specific absorption bands at 246 nm, 292 nm, 614 nm, and 665 nm. The % removal efficacy (RE%) was calculated based on the change in absorption intensity according to Eq. 1.

$$\text{RE}\%\;=\;\text{100}\;\text{x}\;(\frac{\left(I_{\text{0}}-I_{f}\right)}{I_{\text{0}}})$$ (1)

Where $I_{\text{0}}$ is the initial absorption intensity and $I_{f}$ is the final absorption intensity.

The results indicate that the use of pure Fe_3_O_4_ NPs and pure PS powder as adsorbents shows removal efficiency of about 30.28% and 31.69%, respectively. These results confirmed the ability of pure Fe_3_O_4_ NPs and pure PS powder to remove MB. To enhance the removal efficiency, several Fe_3_O_4_/PS nanocomposites containing different percentages of Fe_3_O_4_ (5%, 10%, 20%, and 30%) were fabricated and used for MB removal. The results (**Fig 5b**) demonstrate that the removal efficiency of the Fe_3_O_4_/PS nanocomposites increased with the increase in the percentage of Fe_3_O_4_ until reaching the maximum (67.27%) for the 20% Fe_3_O_4_/PS nanocomposite. It is worth noting that the further increase in the Fe_3_O_4_ percentage (30% Fe_3_O_4_/PS nanocomposite) results in a slight decrease in the removal efficiency (61.09%). The high removal efficiency of the Fe_3_O_4_/PS nanocomposites confirms the synergistic effect between the Fe_3_O_4_ NPs and the PS powder. However, using a large amount of Fe_3_O_4_ NPs could negatively affect the composite’s removal efficiency due to the filling of the PS pores and blocking the active sites of both components. Thus, the 20% Fe_3_O_4_/PS nanocomposite was selected as the adsorbent for the further experiments.

#### 3.2.2. Effect of adsorbent dose on the removal efficacy.

**Fig 6:**
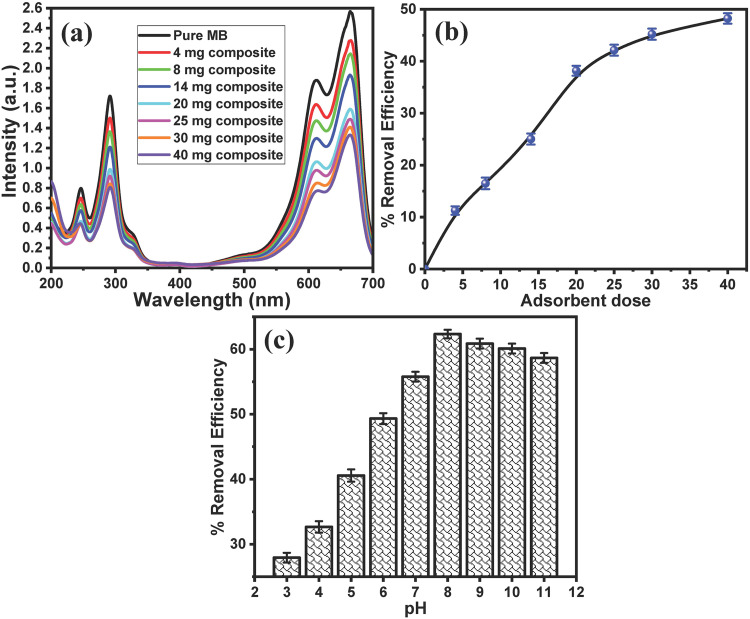
Effect of adsorbent dose and pH on the removal efficacy. (a) UV-vis spectra of 25 mg/L of MB solution before and after treatment with different doses of Fe_3_O_4_/PS composites (from 4 mg to 40 mg) at 25 °C for 15 min, (b) the relationship between the adsorbent dose and the removal efficiency of MB, and (c) the effect of the pH on the removal efficiency.

The adsorbent dose plays a vital role in the adsorbent removal efficacy; several adsorbent doses ranging from 4 mg to 40 mg were used, and their removal efficiency was studied. **Fig 6a** shows the UV-vis spectra of 25 mg/L of MB before and after treatment with different Fe_3_O_4_/PS adsorbent doses. The results indicated that the intensity of the absorption band of the MB solution decreased after treatment with PPy/cellulose, and the UV-vis intensity decreased with increasing adsorbent dose. The correlation between the adsorbent dose and the RE% is represented in **Fig 6b**. The findings demonstrate that the RE% increased with increasing adsorbent dose almost linearly until the adsorbent dose of 25 mg. Then, the RE% slightly increased with increasing adsorbent dose. Furthermore, using 40 mg of the adsorbent could remove over 62% of the MB at room temperature.

#### 3.2.3. Effect of pH on the MB removal.

**Scheme 1:**
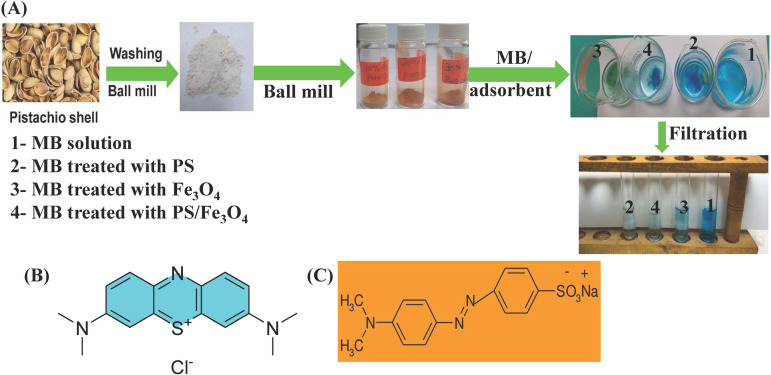
(A) Development of Fe_3_O_4_/PS adsorbent for MB dye removal, (B) Methylene blue dye structure, and (C) Methyl Orange dye structure.

The pH of the dye solution significantly affects the adsorption capacity of the adsorbent materials due to its effects on the adsorbent active sites as well as the dye form [44]. The chemical structure of MB dye (Scheme 1B) demonstrates that MB dye is a cationic dye (basic), which carries a positive charge and exists as a cation in solution. Thus, the solution pH has a remarkable effect on the form of the MB dye. Moreover, it was reported that the surface charge of the Fe_3_O_4_ nanoparticles is tunable and based on the solution pH. The Fe_3_O_4_ nanoparticles would be positively charged in acidic/neutral solutions and negatively charged in basic solutions [45]. The surface charge of the Fe_3_O_4_ NPs was studied by measuring their zeta potential (S8 Fig in S1 File). The results show that the synthesized Fe_3_O_4_ NPs in distilled water have an average zeta potential of +3.16 mV. Thus, the adsorption mechanism is based on the electrochemical force interactions of the dye with the material. The pH effect within the range from 3 to 11 on the MB %RE at Fe_3_O_4_/PS was examined. **Fig 6c** represents the correlation between the pH and the %RE for the treatment of 25 mL of 25 mg/L MB under an adsorbent dose of 15 mg for 15 min, and at a temperature of 298 K. The results reveal that the percentage removal increases as the pH increases until pH = 8. Then, the percentage removal of MB on Fe_3_O_4_@PS decreased with the further increase of the pH. In an acidic solution, the adsorbent’s surfaces are protonated and become positively charged, resulting in electrostatic repulsion forces between the anionic MB molecules and positively charged adsorbent nanocomposites [46,47].

Furthermore, in an acidic medium, competition between the H^+^ ions and the cationic MB molecules occurs for the adsorbent active sites. Increasing the pH value results in the deprotonation of the adsorbent’s surface, making it more negatively charged, which promotes the electrostatic attraction forces between the positively charged MB molecules and the negatively charged adsorbent [48,49]. On the other hand, in a strongly alkaline solution, MB isn’t stable, since cationic MB may react with the hydroxide ions (OH^-^), which results in hydrolysis and cleavage of the MB molecules [50]. Thus, pH 7 was chosen as the ideal value for the initial MB solution, since the treatment of dye wastewater is generally neutral.

#### 3.2.4. Effects of the initial concentration on the MB removal.

One of the factors that influences the dye removal process is the dye’s initial concentration. The effects of several initial concentrations of the dye ranging from 2.50 to 25 mg/L on dye uptake were investigated. A 15 mg amount of the adsorbent was used for the treatment of 25 mL of MB solutions with different concentrations at room temperature.

The influence of MB initial concentration on adsorption was investigated under optimized pH conditions at 25°C. The uptake of MB dye by Fe_3_O_4_/PS nanocomposite was calculated by using Eq. (2) as follows,


$$q_{e}=\;\frac{\left(C_{\text{0}}-C_{e}\right)V}{m}$$ (2)

Where q_e_ is the amount of dye extracted (mg g^-1^), m is the adsorbent mass, V is the solution volume, C_0_ and C_e_ are the initial and equilibrium concentrations of MB dye (mg L^-1^), respectively.

**Fig 7:**
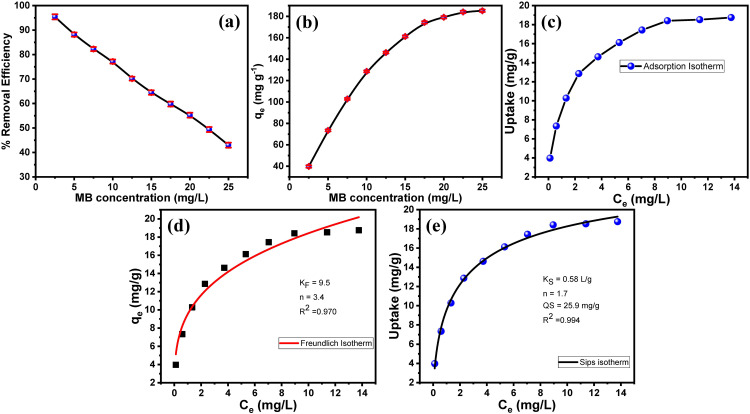
Adsorption kinetics and isotherms. (a) The influence of MB equilibrium concentration on the adsorption capacity of the Fe₃O₄/PS adsorbent, (b & c) present the Freundlich and Sips isotherm models, respectively, for MB adsorption on the Fe₃O₄/PS adsorbent (adsorbent mass of 15 mg, a solution volume of 25 mL, and a contact time of 25 min), (d) Effect of equilibrium concentrations on the adsorption of MB onto the synthesized sorbent, (e) the adsorption isotherm, and (f) Langmuir isotherm C- Sips d- DR e- RP and f Temkin isotherms.

The equilibrium concentration of MB was a critical parameter governing the adsorption capacity of the Fe₃O₄/PS nanocomposite. **Fig 7a** demonstrates the effect of the initial concentration on the dye uptake. The results confirm that the dye uptake (qₑ) linearly increases at lower concentrations (2.50–25 mg/L) due to a high concentration gradient driving mass transfer. At a higher MB initial concentration, the uptake reaches a plateau, suggesting saturation of active adsorption sites. These results confirm that the dye’s initial concentration significantly impacts the adsorption capacity of the adsorbent.

To decipher the nature of this equilibrium, the data were analyzed using several isotherm models.

To further analyze the adsorption behavior, the equilibrium data were fitted with the Freundlich (Eq. 3) and Sips (Eq. 4) isotherm models (Fig 7b and c), both of which provided excellent fits with correlation coefficients (R² > 0.98). The Freundlich model, which assumes multilayer adsorption on heterogeneous surfaces [51], was suitable for describing the adsorption process, supporting the hypothesis of non-uniform binding sites. The Sips model, a three-parameter hybrid of Langmuir and Freundlich isotherms [52,53], further improved the fit quality, suggesting a combination of monolayer and multilayer adsorption mechanisms. The poor fit of the Langmuir model precluded a purely homogeneous, monolayer adsorption mechanism.

Furthermore, applying a third parameter model, such as the Sips model [54], allows for improved quality of the fit.


$$q_{e}=K_{f}C_{e}^{\text{1}/n}\;\;\;\;\;\;\;\;\;\;\;\;\;\;\;\;\;\;\;\;\;\;\;\;\;\;\;\;\;\;\;$$ (3)

$$q_{e}=\frac{Q_{s}K_{s}C_{e}^{n_{s}}}{\text{1}+K_{s}C_{e}^{n_{s}}}$$ (4)

where K_F_ and K_S_ represent adsorption affinity constants (related to adsorption energy), n_F_ and n_S_ are dimensionless heterogeneity factors, and Q_S_ denotes the maximum adsorption capacity in the Sips model.

In contrast, the excellent fits of the Freundlich (R² = 0.970) and Sips (R² = 0.994) models confirmed a complex process on a heterogeneous surface. The Sips model, a hybrid of Langmuir and Freundlich isotherms, provided the best description, with a predicted maximum capacity (Q_s_ = 25.9 mg/g) closely matching experimental data. The exponents from both models (n_F_ = 3.4, n_S_ = 1.6), being greater than 1, initially suggested a high-affinity, chemisorption-dominated process.

This interpretation was refined using additional models.


$$q_{e}=\frac{{K_{RP}C}_{e}}{\text{1}+aC_{e}^{\beta }\;}$$ (5)

where qₑ is the equilibrium adsorption capacity (mg/g) and Cₑ is the equilibrium concentration (mg/L). The constants k_r_ (L/g) and a_r_ (L/mg)^β^ relate to capacity and affinity, while the exponent β (0 < β < 1) indicates surface heterogeneity.

The Dubinin−Radushkevich (D-R) isotherm is a temperature-dependent model used to estimate the apparent energy of adsorption (**Fig 7d**). The model is represented by the following equations


$$q_{e}={\text{Q}}_{e}\;e^{-k\epsilon^{\text{2}}}$$ (6)

$$\epsilon =\text{RT}\;\text{ln}(\;\text{1}+\frac{\text{1}}{C_{e}})$$ (7)

where ε = RT ln(1 + 1/Ce), qs is the theoretical isotherm saturation capacity (mg/g), K is the D-R isotherm constant (mol^2^/kJ^2^), ε is the D-R isotherm constant, R is the gas constant, and T is the absolute temperature (K).

The excellent fit of the Redlich-Peterson (R-P) isotherm (R² = 0.988) with an exponent (β = 0.82) close to 1 reinforced the Sips model conclusion, indicating a system leaning towards monolayer coverage on a heterogeneous surface (**Fig 7e**). Furthermore, the Temkin isotherm (R² = 0.981) supported the presence of a heterogeneous surface, with its parameters indicating a strong adsorbent-adsorbate affinity (α = 20.5 L/mg). However, a critical insight was provided by the Dubinin-Radushkevich (D-R) isotherm, which yielded a mean free energy of adsorption (E) of 0.65 kJ/mol. This value, along with the analogous adsorption energy of 0.73 kJ/mol derived from the Temkin β constant, is unequivocally within the range characteristic of physisorption (E < 8 kJ/mol). In conclusion, the adsorption equilibrium is most accurately described by the Sips and R-P models. The process is a high-affinity, heterogeneous adsorption where physisorption is the dominant mechanism. The seemingly chemisorptive indicators from the Freundlich and Sips exponents are reconciled by the highly heterogeneous surface of the Fe₃O₄/PS nanocomposite, which creates strong, specific binding sites that enhance the physisorption interaction, leading to the observed high affinity and complex equilibrium behavior.

In addition, Temkin equation represents as follows Eq. (8):


$$q_{e}=\beta \;\text{ln}(\alpha \;C_{e})$$ (8)

The adsorption equilibrium data exhibited an excellent fit to the Temkin isotherm model (**Fig 7f**), as confirmed by the high correlation coefficient (R² = 0.981). This strong agreement suggests that the heat of adsorption decreases linearly with coverage, a characteristic of heterogeneous surfaces where adsorbate-adsorbate interactions are significant. The model parameters provide further mechanistic insight: a high equilibrium binding constant (α = 20.5 L/mg) indicates a strong affinity between MB and the adsorbent surface. Furthermore, the adsorption potential (β = 3.4) corresponds to a mean adsorption energy of approximately 0.73 kJ/mol, calculated from B = RT/β. This low energy value, significantly less than 8 kJ/mol, strongly implies that the adsorption process is predominantly physisorptive in nature. In summary, the Temkin model effectively describes the adsorption system, revealing a process governed by strong adsorbent-adsorbate affinity on a heterogeneous surface, yet driven primarily by physical forces such as electrostatic interactions.

#### 3.2.5. Contact time effect on the MB removal.

**Fig 8:**
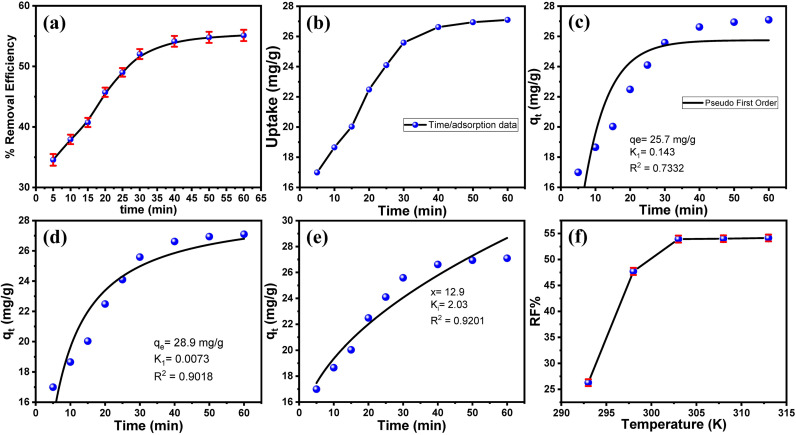
The factors affecting the adsorption. (a) Effect of contact time on MB removal efficiency by the Fe₃O₄/PS adsorbent. Part (b) the changes in the adsorption capacity of MB over time at pH 7. The nonlinear adsorption kinetics models, (c) pseudo-first-order, (d) pseudo-second-order, (e) Intraparticle diffusion plots, and (f) the effect of temperature on MB adsorption using the Fe₃O₄/PS adsorbent.

Furthermore, contact time plays an essential role in the removal efficiency, so the effect of treatment time on removal efficiency was also studied. **Fig 8a** shows the effect of the treating of 25 mg/L of MB with 15 mg of the adsorbent over different periods (0–60 min). The results confirmed that the removal percentage of MB dye increased almost linearly within the first 20 min, rising from 40.78% at 5 min to 53.96%, indicating an initial fast adsorption phase likely driven by surface binding and film diffusion. The rate of adsorption gradually slowed thereafter, reaching 63.88% at 40 minutes and eventually plateauing near 65.03% by 60 minutes, suggesting saturation of the active sites and attainment of equilibrium. This trend aligns with typical adsorption kinetics, where an initial rapid uptake is followed by slower intraparticle diffusion and eventual stabilization. The near-constant RE% beyond 40 minutes implies that the composite’s maximum adsorption capacity under these conditions was achieved, highlighting the importance of contact time optimization for efficient MB removal. The data further support the earlier proposed hybrid mechanism, where both surface adsorption and pore diffusion contribute to the overall process.

The kinetic data was analyzed using nonlinear forms (Fig 8b–e) of PFOR (Eq. 9) and PSOR (Eq. 10):


$$q_{t}=q_{e}\;\;(\text{1}-e^{-kt})\;\;\;\;\;\;\;\;\;\;\;\;\;\;\;\;\;\;\;\;\;\;\;$$ (9)

$$q_{t}=\frac{k_{\text{2}}q_{e}^{\text{2}}t}{\text{1}+k_{\text{2}}q_{e}t}\;\;\;\;\;\;\;\;\;\;\;\;\;\;\;\;\;\;\;\;\;\;\;\;\;\;\;\;\;\;\;\;\;\;\;\;\;\;\;\;\;\;\;$$ (10)

Here, k₁ (min⁻¹) and k₂ (g/mg·min) denote the rate constants, while qₜ (mg/g) represents the time-dependent adsorption capacity. The PSOR model exhibits a marginally better fit (R² = 0.901) to the experimental data compared to PFOR (R² = 0.733). This suggests that chemisorption, likely through coordination bonding at nitrogen-containing active sites, plays a dominant role in the adsorption process. However, the close agreement between the PFOR-predicted equilibrium capacity (25.7 mg/g) and the experimental value (26.6 mg/g) suggests that physical adsorption also contributes, indicating a hybrid adsorption mechanism. The adsorption kinetics followed a three-stage process: an initial rapid phase where MB molecules quickly migrated to the composite surface, followed by a slower intraparticle diffusion phase through the porous structure, and finally a saturation stage where stable [composite→MB]ⁿ⁺ complexes formed via coordination bonds.

Further investigation using the Weber-Morris intraparticle diffusion model (Eq. 11) revealed that pore diffusion was the rate-limiting step, supported by a strong correlation (R² = 0.92) between the model and experimental data.


$$q_{t\;}\;=x+\;k_{i\;}t^{\text{0}.\text{5}}$$ (11)

where *x* represents boundary layer thickness and *kᵢ* (2.03) denotes the intraparticle diffusion rate constant (12.9 mmol/g·min⁰·⁵).

The non-zero interception in the diffusion plot indicated some influence of boundary layer effects during the initial adsorption stages, suggesting that film diffusion also played a role. This behavior is consistent with the composite’s hierarchical pore structure, where the initial rapid adsorption corresponds to surface binding, followed by gradual diffusion through mesopores, and eventual equilibrium through pore filling and chemical complexation. The findings highlight a dual adsorption mechanism involving both physical and chemical interactions, with implications for optimizing the composite’s design, such as enhancing mesoporosity to improve diffusion rates or increasing nitrogen-active sites to strengthen chemisorption. Future studies could explore mixed kinetic models to better capture the observed hybrid adsorption behavior.

#### 3.2.6. Effects of treatment temperature on the MB removal.

Due to the crucial role of temperature in both physical and chemical adsorption processes, the effect of the treatment temperature (293 K to 313 K) was studied as shown in Fig 8f. 25 mL of each MB (25 mg/L) solution was treated with 15 mg of the adsorbent at different temperatures (298–313 K) for 15 min. The RF% increases sharply from 26.28% at 293 K to 47.7% at 298 K, indicating a strong temperature dependence, likely due to enhanced kinetic energy or reaction rates as described by the Arrhenius equation. Beyond 303 K, the R_F_% plateaus near 54%, suggesting a limiting factor such as catalyst saturation, equilibrium constraints, or complete degradation of the reactive species. This plateau implies that further temperature increases do not significantly improve efficiency, possibly due to the exhaustion of active sites or thermodynamic equilibrium.

#### 3.2.7. Selectivity, recyclability, and real sample application.

**Fig 9:**
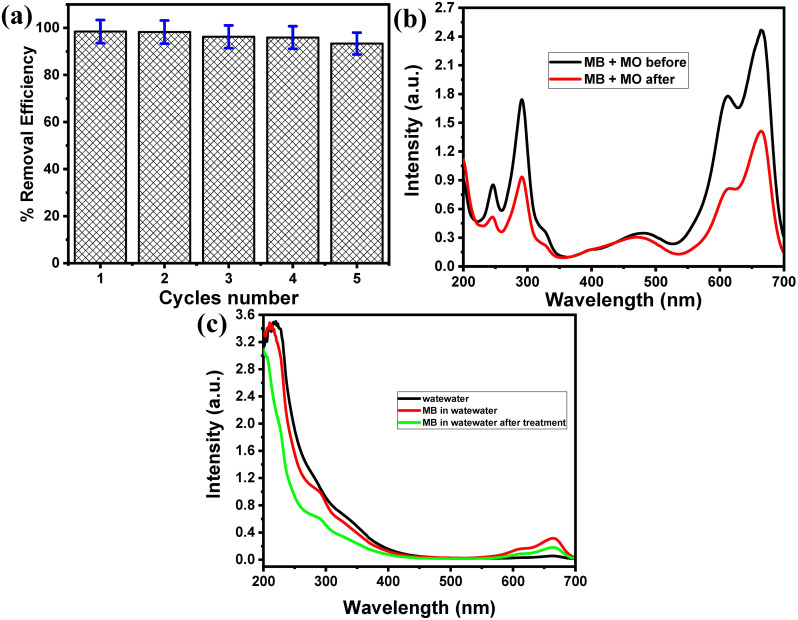
Real sample application. (a) Reusability studies of 100 mg of Fe_3_O_4_/PS composites in terms of removal efficiency changes for the capturing properties of 15 mg/L of MB after five regeneration/reuse cycles, (b) Selectivity of Fe_3_O_4_/PS composite toward MB in the presence of the MO ionic dye, and (c) monitoring of MB in industrial wastewater samples.

The high stability and reusability of adsorbent materials are another important issue during the development of inexpensive adsorbents, particularly for practical applications. Thus, the investigation of the adsorbent regeneration is required. The reusability of the Fe_3_O_4_/PS adsorbent towards MB was investigated, which is essential to evaluate its industrial utilization and cost-effectiveness. The Fe_3_O_4_/PS adsorbent was regenerated after MB adsorption studies by desorbing MB using ethanol. The MB@Fe_3_O_4_/PS mixture was ultrasonicated for 10 min, washed repeatedly three times with distilled water, filtered, and dried in an oven at 60 °C for 3 h, then reused for the next adsorption-desorption cycle. Fig 9a shows the adsorptive performance of the Fe_3_O_4_/PS adsorbent towards MB during five successive cycles. The MB removal efficacy in the first cycle was 98.48%. Notably, the removal efficacy decreased to 93.36%. These results demonstrate that the adsorbent retains more than 6% of its original removal efficiency after five cycles. Thus, due to the high stability and high percentage removal for MB adsorption, the Fe_3_O_4_/PS adsorbent is a sustainable choice for the remediation of wastewater containing dyes.

The development of highly selective adsorbent materials with high adsorptive performance for the simultaneous adsorption of cationic and anionic dyes from complex wastewater is urgently needed. The adsorptive performance of Fe_3_O_4_/PS adsorbent towards MB cationic dye and MO anionic dye (Scheme 1C) were examined. **Fig 9b** (black curve) shows the UV-vis spectrum of a mixture of 25 mg/L of MB and MO dyes, which demonstrates the appearance of a new absorption band at 481 nm that is characteristic of MO dye. The UV-vis spectrum of the MB and MO mixture after treatment with 15 mg/L of Fe_3_O_4_/PS adsorbent at room temperature for 15 min is represented in **Fig 9b (red curve)**. The results demonstrate that the treatment of the MB and MO mixture results in the %RF of 46.93% and 12.46% for MB and MO, respectively. The high affinity of the Fe_3_O_4_/PS adsorbent towards MB in comparison to MO is related to the electrostatic attraction force with the cationic dye and the electrostatic repulsion with the anionic dye [55]. These results indicate the high adsorption selectivity of the Fe_3_O_4_/PS adsorbent towards cationic MB.

To examine the practical applicability of Fe_3_O_4_/PS adsorbent in real wastewater samples, an industrial wastewater sample was obtained from Al-Juhfah, Rabigh, KSA. Firstly, we filtered off the sample to remove any solid contaminants. Then, the filtrate was used to investigate the adsorptive performance of the developed adsorbent for MB removal in a complicated matrix. The UV-vis spectrum of the wastewater sample is represented in **Fig 9c (black curve)**, which shows a strong absorption band in the UV wavelength region (200–250 nm) that is related to the presence of many salts. A 10 mg/L of MB was spiked in the wastewater sample, and its UV-vis spectrum was recorded (**Fig 9c, red curve**). The UV-vis spectrum shows the characteristic set of MB absorption bands. The spiked sample was then treated with 10 mg of Fe_3_O_4_/PS adsorbent at room temperature for 15 min. **Fig 9c (green curve)** shows the UV-vis spectrum of the spiked sample after treatment, which confirms the ability of the developed adsorbent for MB removal in industrial wastewater samples.

Finally, the superiority of 20% Fe_3_O_4_/PS nanocomposite over all tested samples is consistent with the data obtained from the SEM images, which clarify that the excessive Fe₃O₄ may block essential pores, slightly reducing adsorption performance, as seen for the 30% composite. The morphological features, such as increased roughness and homogeneous nanoparticle coating, directly contribute to the composite’s superior performance in methylene blue removal and stability during repeated use.

## 4. Conclusions

Four Fe₃O₄/pistachio shell (PS) nanocomposites with varying Fe₃O₄ content were successfully synthesized via a mechanochemical ball-milling method and thoroughly characterized by SEM, FTIR, and EDX. The composite with 20% Fe₃O₄ demonstrated the highest methylene blue (MB) removal efficiency of 67.27%, while the overall optimized conditions (2.5 mg/L MB concentration, 15 mg adsorbent dose, 15 min contact time, 25 °C) achieved a maximum removal efficiency of 95%. Adsorption isotherm analysis revealed that the Sips model best described the equilibrium data with a maximum adsorption capacity of approximately 25.9 mg/g, indicating a hybrid physisorption-chemisorption mechanism. The composite showed selective adsorption for cationic MB over anionic dyes and maintained high removal efficiency (above 93%) over five regeneration cycles. Application to industrial wastewater confirmed its practical viability. These results demonstrate the potential of Fe₃O₄/PS composites as cost-effective, sustainable, and reusable adsorbents for efficient dye removal in water purification applications.

## Supporting information

S1 File Characterizations of the fabricated adsorbents. (DOCX)
