# Supplementary material for: Harnessing agro-waste for the high-efficiency removal of methylene blue using ball-milled magnetic Fe3O4@pistachio shell composites: From waste to resource
Source: PLoS One. 2025 Nov 25;20(11):e0337235. doi: 10.1371/journal.pone.0337235 (PMC12646412; doi:10.1371/journal.pone.0337235)
Supplement: S1 File — (DOCX) [file pone.0337235.s001.docx]

Harnessing Agro-Waste for the High-Efficiency Removal of Cationic Dyes using Iron Oxide/Pistachio Shell Composites

Tamer S. Saleh^a,*^, Mohamed N. Gomaa^b^, Abdullah Akhdhar^a^, Abdullah Saad Al‐Bogami^a^, Waleed A. El-Said^a,*^

^a^*Department of Chemistry, College of Science, University of Jeddah, P.O. 80327, Jeddah 21589, Saudi Arabia*

^b^*Department of Biology Science, College of Science, University of Jeddah, P.O. 80327, Jeddah 21589, Saudi Arabia*

*Corresponding author: ([tssayed@uj.edu.sa](mailto:tssayed@uj.edu.sa), [waahmed@uj.edu.sa](mailto:waahmed@uj.edu.sa))

**Supplementary Materials**


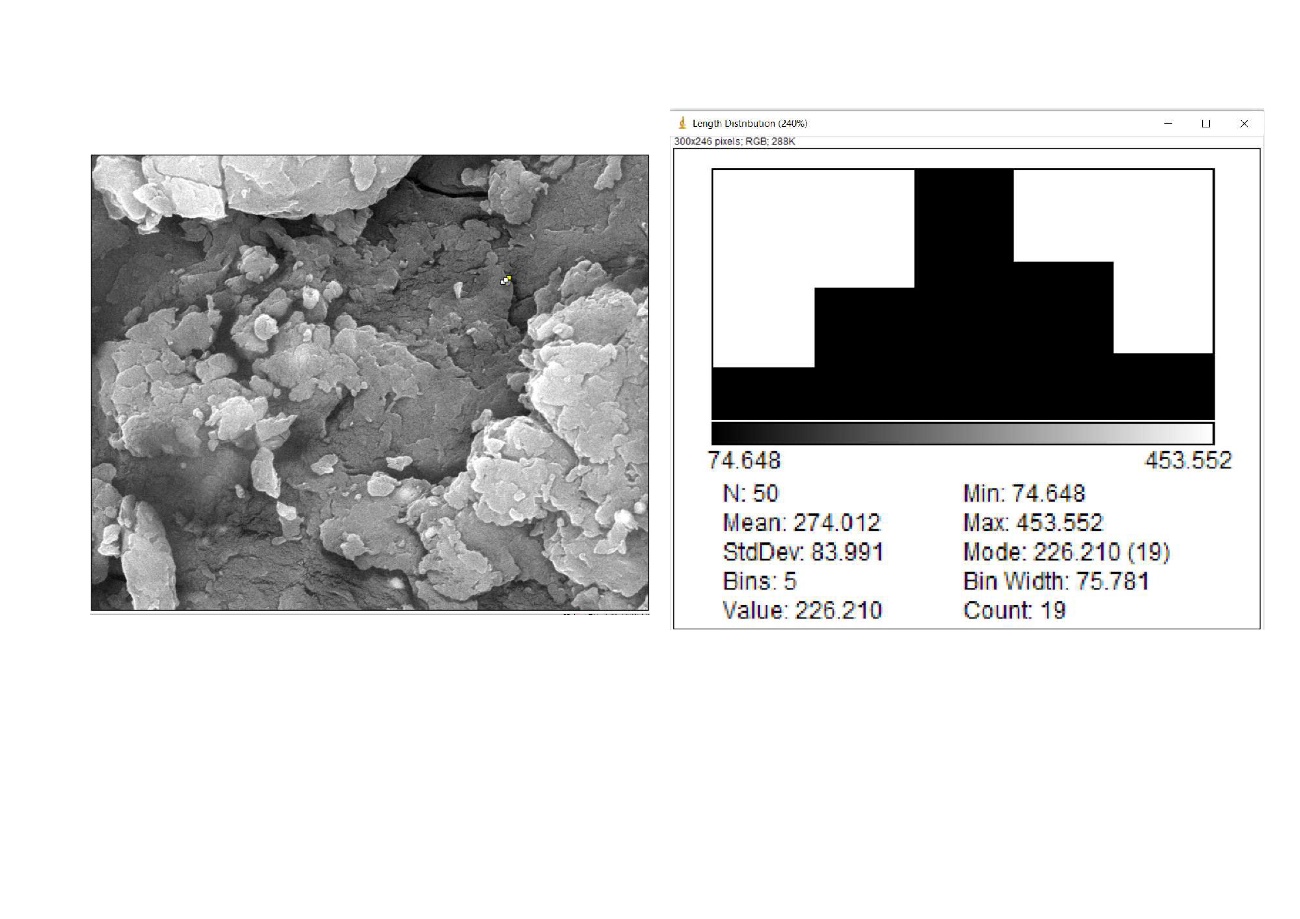


**Fig S1.** Diameter distribution of 45 Fe_3_O_4_ Particles


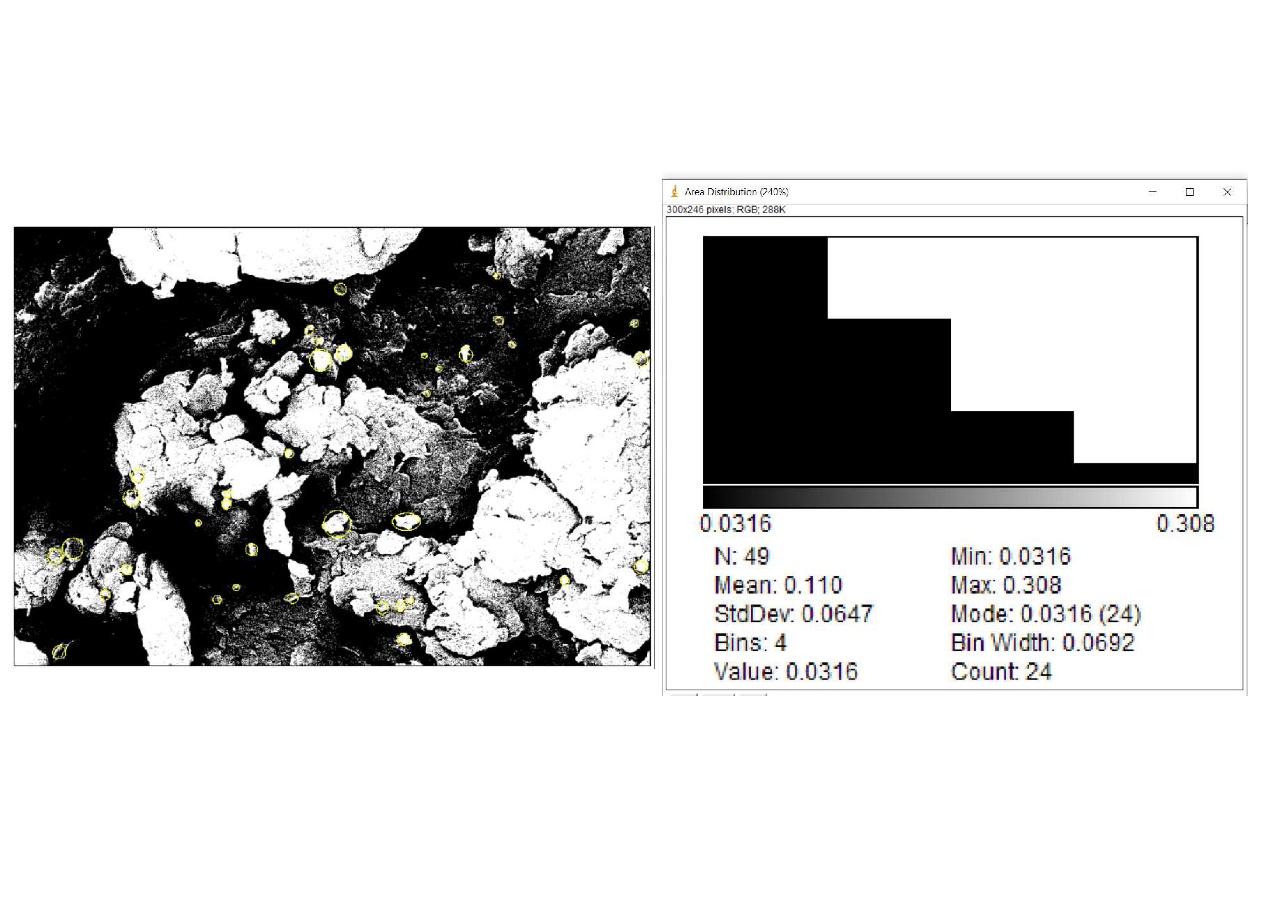


**Fig S2.** Area distribution of 45 Fe_3_O_4_ Particles


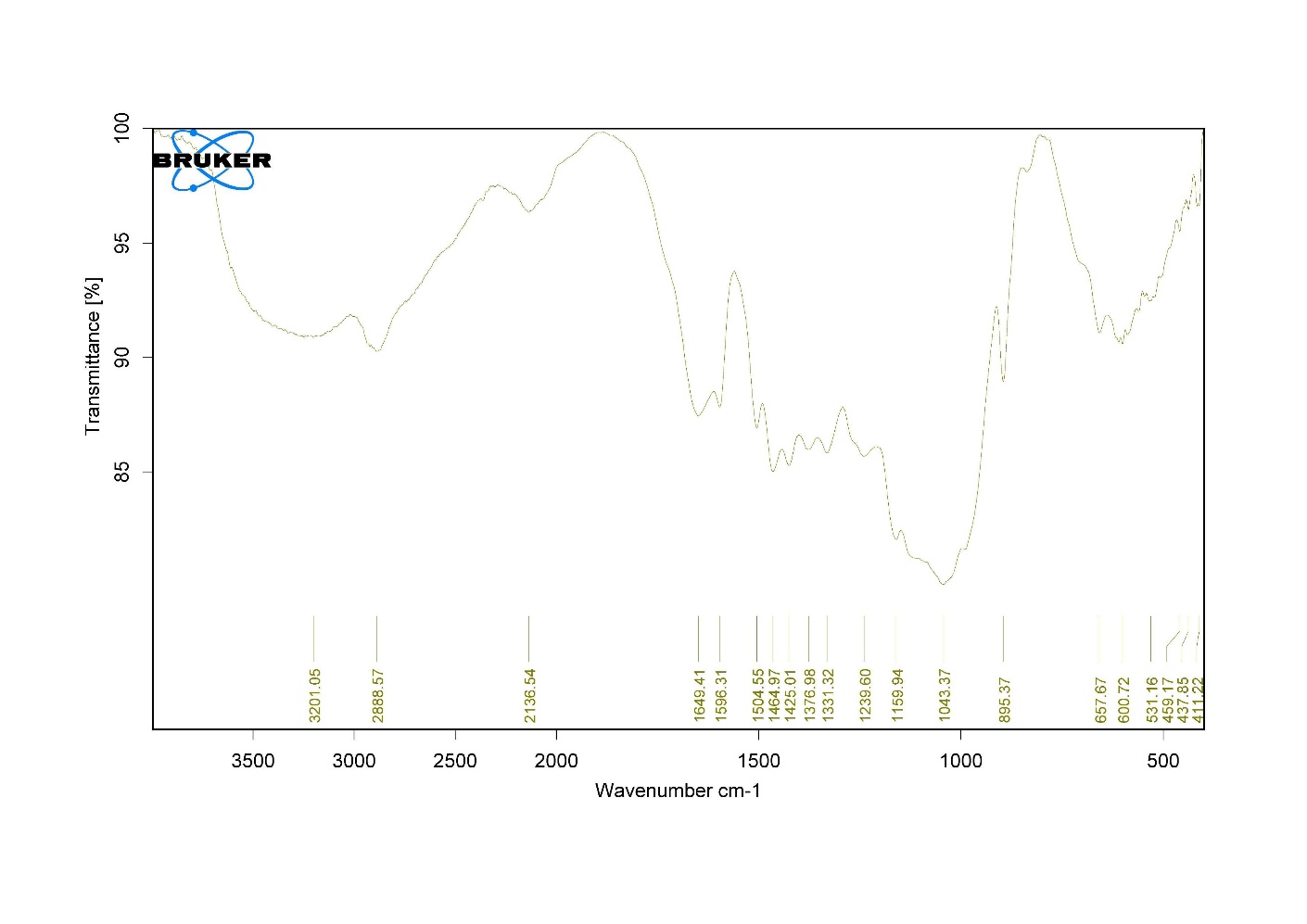


**Fig S3.** FTIR of PS powder


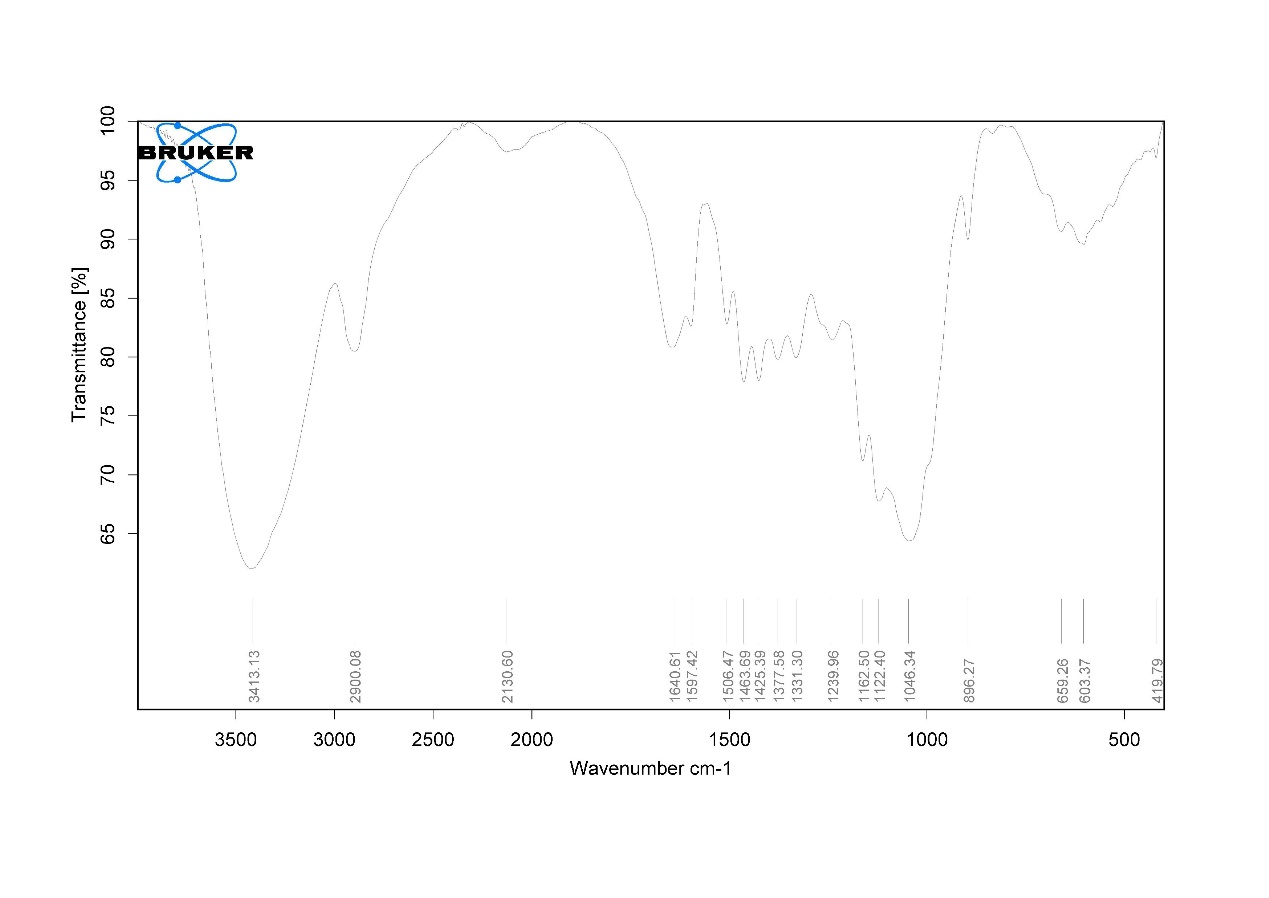


**Fig S4.** FTIR of 5% Fe_3_O_4_/PS


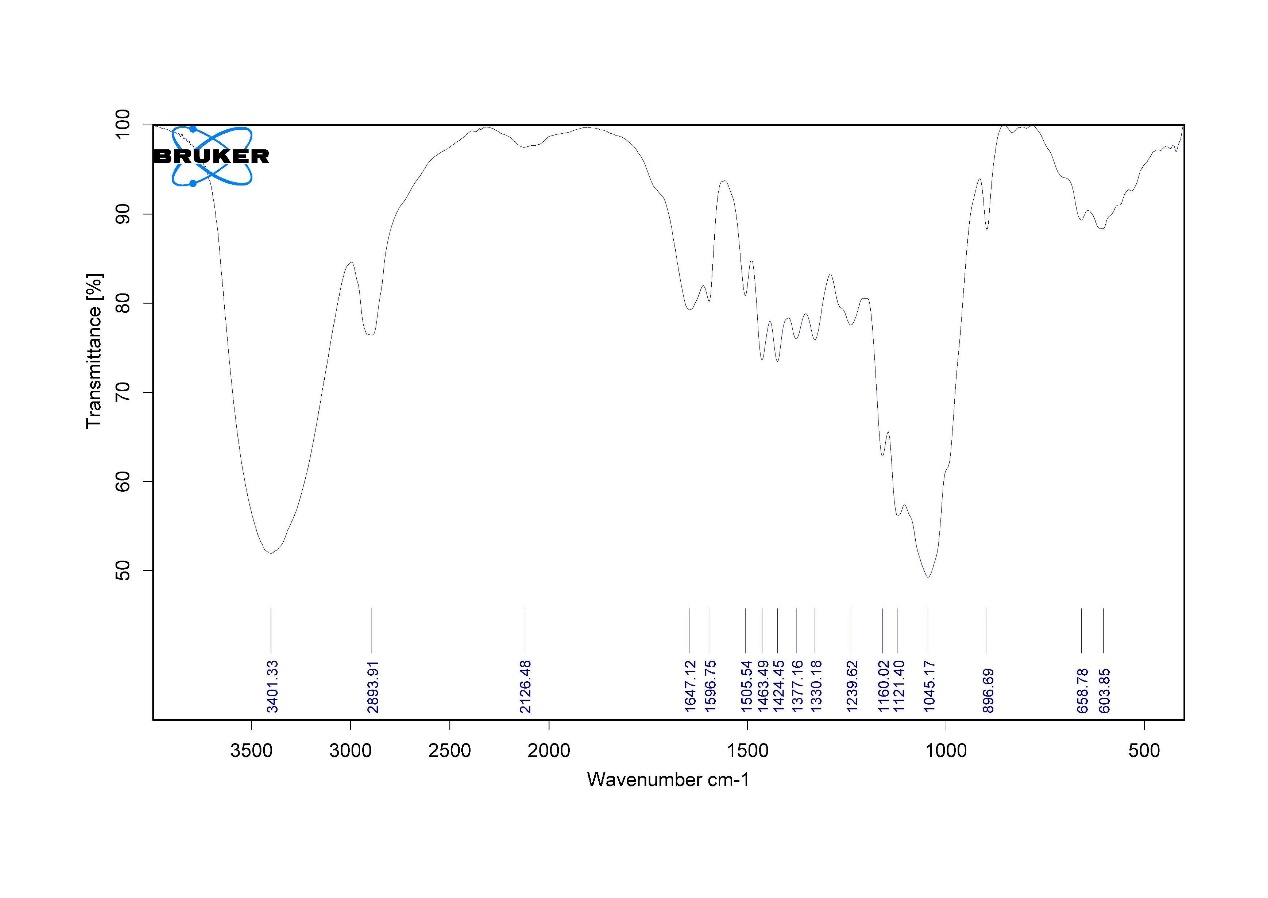


**Fig S5.** FTIR of 10% Fe_3_O_4_/PS


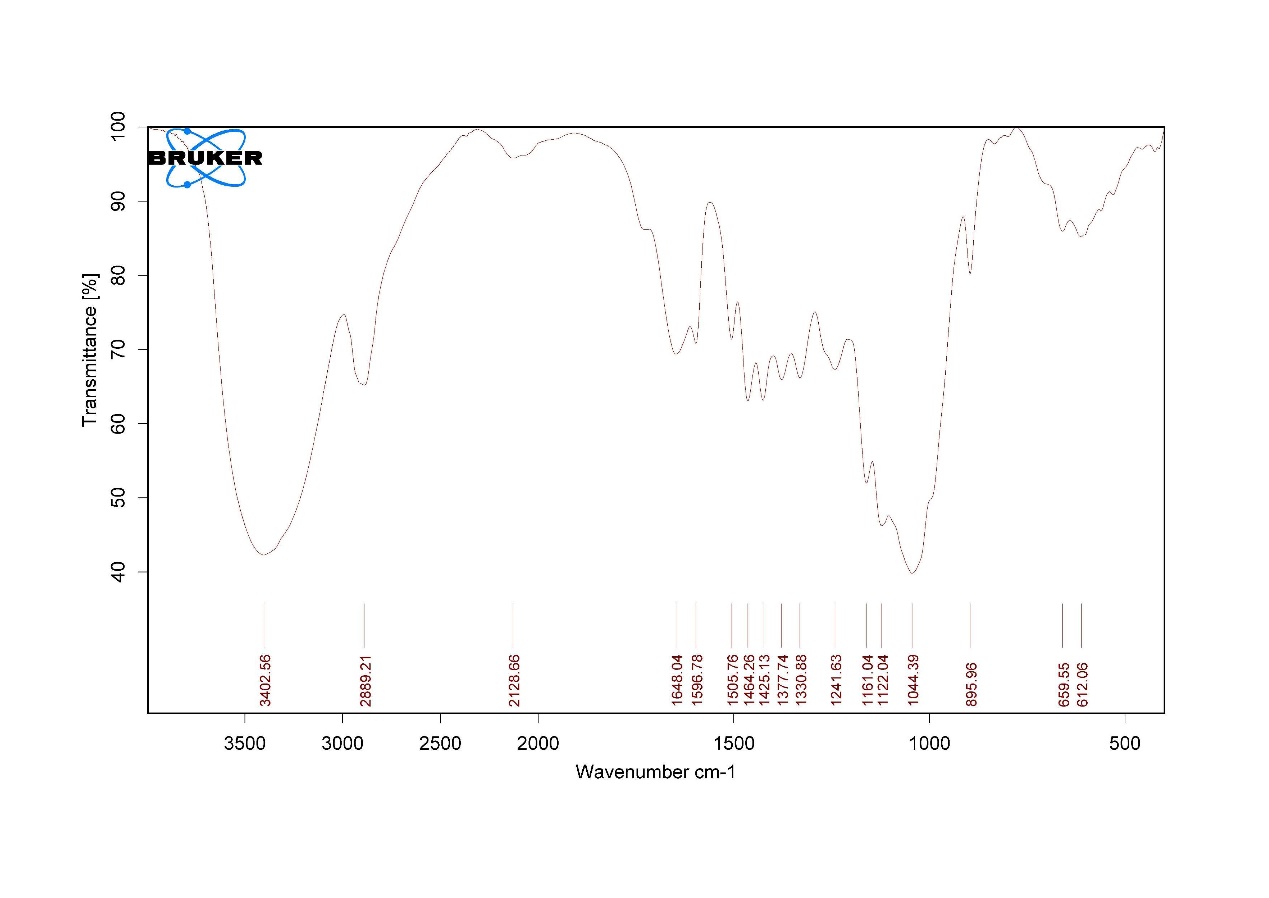


**Fig S6.** FTIR of 20% Fe_3_O_4_/PS


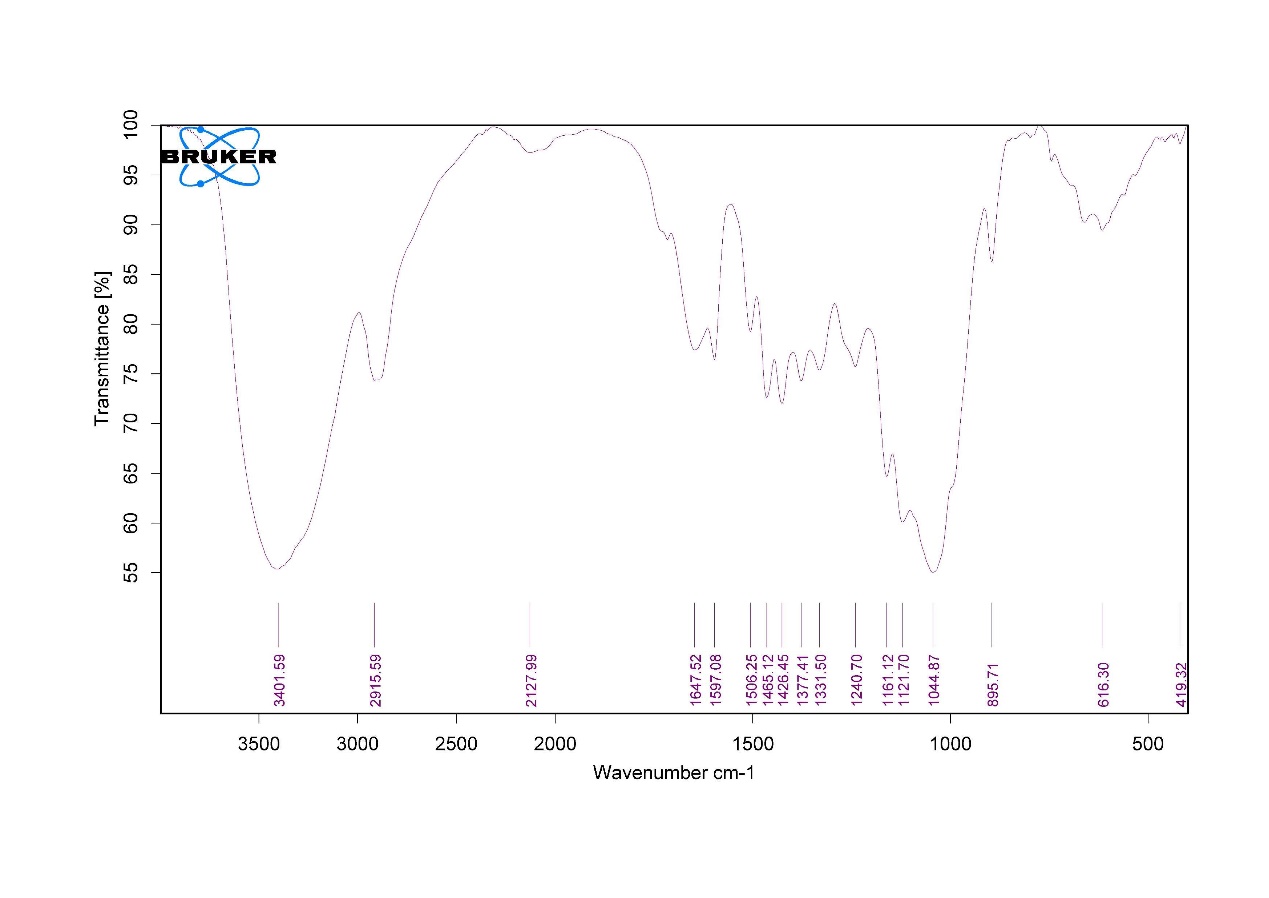


**Fig S7.** FTIR of 30% Fe_3_O_4_/PS


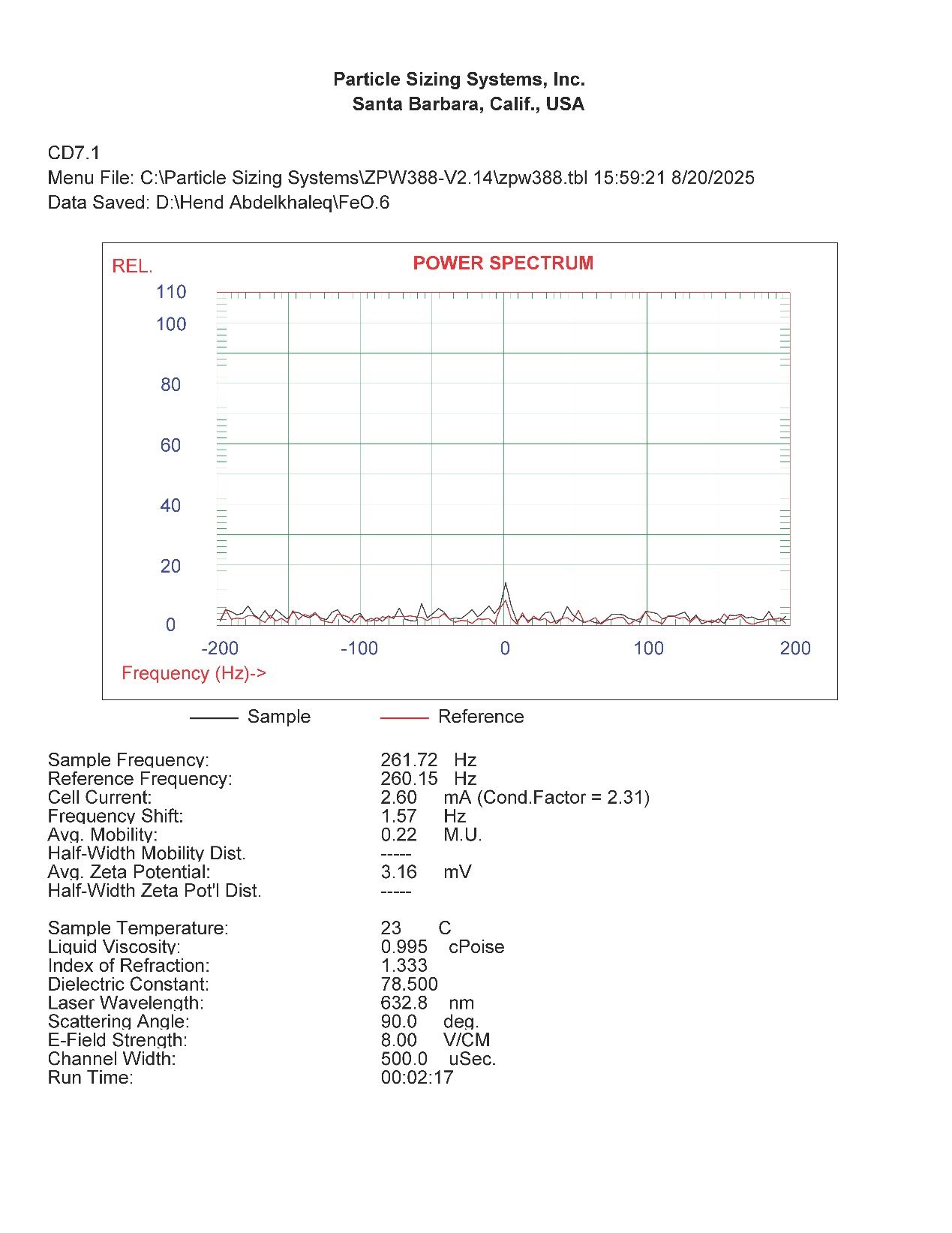


**Fig S8.** Zeta potential of Fe_3_O_4_ NPs
